# Quality of Community Pharmacy Practice in Antibiotic Self-Medication Encounters: A Simulated Patient Study in Upper Egypt

**DOI:** 10.3390/antibiotics8020035

**Published:** 2019-04-01

**Authors:** Abdullah I. Abdelaziz, Abdelrahman G. Tawfik, Khaled A. Rabie, Mohamad Omran, Mustafa Hussein, Adel Abou-Ali, Al-Shaimaa F. Ahmed

**Affiliations:** 1 Department of Pharmaceutics, Faculty of Pharmacy, Minia University, Minia 61519, Egypt; abdullahislam.mu@gmail.com; 2 Department of Pharmacology and Toxicology, Faculty of Pharmacy, Deraya University, Minia 61512, Egypt; abdelrahman.gamal@deraya.edu.eg; 3 Medical Representative, Novartis 61519, Egypt; khalid.ahmed.rabie@gmail.com; 4 Community Pharmacist, Minia 61519, Egypt; muhammad.o.yusef@gmail.com; 5 Joseph J. Zilber School of Public Health, University of Wisconsin-Milwaukee, Milwaukee, WI 53201, USA; husseimh@uwm.edu; 6 Global Safety Officer at Sanofi Pasteur, Toronto Area, ON M2R 3T4, Canada; 7 Department of Pharmacology and Toxicology, Faculty of Pharmacy, Minia University, Minia 61519, Egypt; shaimaa.faissal@minia.edu.eg

**Keywords:** simulated patient study, antibiotic misuse, pharmacy practice, common cold, acute bronchitis, Egypt

## Abstract

Antibiotic misuse, either by patients or healthcare professionals, is one of the major contributing factors to antimicrobial resistance. In many Middle Eastern countries including Egypt, there are no strict regulations regarding antibiotic dispensing by community pharmacies. In this study, we examined antibiotic dispensing patterns in Egyptian community pharmacies. About 150 community pharmacies were randomly chosen using convenience sampling from the five most populous urban districts of Minia Governorate in Egypt. Two simulated patient (SP) scenarios of viral respiratory tract infection requiring no antibiotic treatment were used to assess the actual antibiotics dispensing practice of. Face-to-face interviews were then conducted to assess the intended dispensing practice. Descriptive statistics were calculated to report the main study outcomes. In 238 visits of both scenarios, 98.3% of service providers dispensed amoxicillin. Although stated otherwise in interviews, most pharmacy providers (63%) dispensed amoxicillin without collecting relevant information from presenting SPs. Findings showed high rates of antibiotic misuse in community pharmacies. Discrepancies between interviews and patient simulation results also suggest a practice‒knowledge gap. Corrective actions, whether legislation, enforcement, education, or awareness campaigns about antibiotic misuse, are urgently needed to improve antibiotic dispensing practices in Egyptian community pharmacies.

## 1. Introduction

Antibiotics have saved countless lives since their discovery in the 20th century [1]. They have gained more importance in developing countries, where infectious diseases remain among the leading causes of death [2]. Misuse of antibiotics, especially in the treatment of acute upper respiratory tract infections, either by healthcare providers through unnecessary prescribing and dispensing or by self-medicating patients, is considered a major cause of antimicrobial resistance [3,4,5,6], with serious worldwide health and economic consequences [7]. Furthermore, the misuse of antibiotics is associated with increased incidence of adverse reactions of antibiotics in the population [8]. Antimicrobial resistance has also become a portent of a post-antibiotic era where minor infections that were curable for decades could become fatal [9,10,11].

Community pharmacies in several low- and middle-income countries are considered the first point of contact for patients to seek medical and wellness advice [12,13,14]. In such countries, the majority of the population seeks immediate health advice from community pharmacists rather than physicians for many reasons including accessibility, lower cost and less time-consuming services [15,16]. Reports from several Middle Eastern countries have suggested a strong association between the quality of community pharmacy practice and antibiotic misuse [17,18,19,20]. In 2014, a qualitative study conducted in Syria reported that participating pharmacists dispensed antibiotics regularly without asking the patients for prescription. Moreover, most of the participating pharmacists believed that dispensing antibiotics without a prescription had no negative impact on public health [17]. The situation was not much different in Saudi Arabia, where a study in Riyadh reported that most participating community pharmacies not only dispensed antibiotics without a prescription but also in clinical cases where antibiotics were not indicated [18]. The findings of this study suggest that such practices might be due to inadequate training of pharmacists in the recognition and rectification of antibiotic misuse. Another reason for antibiotic misuse could be staffing of pharmacies. Anecdotal and research evidence [21] suggests that pharmacies in Middle Eastern countries are often staffed by non-pharmacists (e.g., salesmen, assistants) who lack proper training in handling antibiotic dispensing and clinical issues in general.

In Egypt, there are no strict regulations on the dispensing of antibiotics, nor are they listed as prescription-only medications (POM). Moreover, the Egyptian Pharmacy Law continues to offer little guidance on the clinical or counseling aspects of pharmacy practice, leaving these important functions essentially unregulated. Thus, dispensing and counseling patients about antibiotics are often carried out without proper training, with their quality entirely dependent on the experiences, attitudes, and initiative of individual pharmacy staff.

In 2011, Dooling et al. conducted a series of in-person surveys and in-depth interviews in Upper Egypt to assess antibiotic misuse in community pharmacy settings [22]. They observed high rates of potentially unnecessary antibiotics dispensing and prescribing for acute respiratory tract infections. While these findings provide a useful baseline, they are limited by social desirability bias where surveyed providers may conceal their true practices to be viewed more favorably by the surveyors.

In this study, we used a simulated patient (SP) approach, the first of its kind to be conducted in this area of Egypt, to investigate the patterns of dispensing antibiotics for self-medication in a random sample of community pharmacies in Upper Egypt. The simulated patient (SP) methodology offers the advantage of overcoming bias due to the Hawthorne effect, where individuals may modify their behavior because of being observed [23]. We used two simulated cases where antibiotic self-medication is highly prevalent: common cold and acute bronchitis.

## 2. Results

### 2.1. Patient Simulation

SP visits were conducted only in pharmacies where amoxicillin was available. The acute bronchitis scenario was performed in 125 pharmacies and the common cold scenario was performed in 113 pharmacies. All pharmacy and pharmacy staff characteristics are reported in Table 1.

Amoxicillin, the antibiotic requested by presenting SPs, was dispensed in almost all of the SP visits; 97.6% of acute bronchitis visits and 99.1% of common cold visits (Table 2). The majority of service providers dispensed amoxicillin without collecting any information about the clinical condition of the patient requesting the drug (63.2% of the acute bronchitis SP visits’ service providers and 62.8% of the common cold SP visits’ service providers). None of the service providers initiated patient consulting with the SP until they were asked for information by the SP. In only few encounters (3.2% of the acute bronchitis SP visits and 3.5% of the common cold SP visits), SPs were asked about medical diagnosis or prescription to dispense Amoxicillin for acute bronchitis symptoms or common cold respectively. However, most of them dispensed amoxicillin despite their awareness of SP condition. In both scenarios, four pharmacists refrained from dispensing. Three of them did that because of penicillin allergy, while one told SP that antibiotics were not indicated for such a case. In the acute bronchitis scenario, 114 (93.4%) pharmacies dispensed Amoxicillin only while the remaining eight (6.6%) pharmacies also dispensed one of the following over-the-counter (OTC) medications: cough preparation, expectorant, bronchodilator, mucolytic, oral decongestant, and analgesic. In the common cold scenario, four pharmacies dispensed one of the following OTC products in addition to amoxicillin: analgesic, antipyretic and oral decongestant. In none of these cases did pharmacists provide the SP with proper medication counseling.

### 2.2. Interview

Out of 100 planned interviews, 83 pharmacies agreed to participate. In 59 (71.1%) of those pharmacies, a pharmacist was interviewed, while a pharmacy technician/assistant was interviewed in the 24 (28.9%) remaining participating pharmacies. Table 3 lists pharmacy and staff characteristics.

The majority of interviewed pharmacists reported that they often or always asked patients about their symptoms (66.1%), symptoms duration (50.8%), and problem history (86.4%) (Table 4). Only 33.9% of the participating pharmacists confirmed that they asked the patients if they had previous medical diagnosis or a prescription before dispensing antibiotics. When asked if antibiotics are indicated for acute bronchitis or common cold infections, the responses of the participating pharmacists varied. While 47.5% of the participants disagreed or strongly disagreed that an antibiotic was essential for treatment of such infections, 35.5% agreed or strongly agreed that an antibiotic was indicated for such cases.

Almost all interviewed pharmacists (96%) either agreed or strongly agreed that antimicrobial resistance is a major public health threat facing the Egyptian population. 64% suggested that irrational dispensing/prescribing is the main cause of antibiotics misuse (Table 5).

## 3. Discussion

This study is the first of its kind in Egypt. We sought to assess antibiotic dispensing patterns in community pharmacy settings using a simulated patient methodology. Although several reports have discussed pharmacists’ antibiotics misuse in different countries that have similar circumstances to Egypt [24,25], studies of antibiotic dispensing practices in Egypt are not commensurate, neither in quality nor quantity, with the precarious situation of antibiotic misuse and resistance rates in Egypt [22,26]. Egypt is ranked among the top three low- and lower-middle-income countries in terms of antibiotic consumption from 2000 to 2015 [27]. Between 2016 and 2018, the World Health Organization (WHO) launched a global program that involved workshops to increase the awareness about antimicrobial resistance and to encourage countries to start their own national surveillance systems of antimicrobial consumption. Among the eight Middle Eastern countries, including Egypt, that have participated in the WHO’s training workshops, only three (Iran, Jordan and Sudan) have provided data regarding antibiotic consumption [28]. Therefore, our study aims to provide the local and international public health community with more insight about the current scenario of antibiotic dispensing in Egypt.

From our analysis, two main findings emerged. First, in nearly all (~99%) SP visits, antibiotics were inappropriately dispensed. Interview results regarding antibiotic dispensing patterns consistently underestimated the more objective levels revealed in SP encounters. Second, information relevant to dispensing and counseling decision was collected in only few encounters.

The discrepancy between SP and interview results regarding antibiotic dispensing patterns clearly demonstrate how self-reported practices [22,26,29] can greatly vary from the real-world practice, revealed using patient simulation methodology. Such discrepancies can be attributable to social-desirability bias (participants tend to provide more socially favorable answers rather than the true ones) as well as the phenomenon of an intention‒behavior gap (where intentions do not always manifest in actions) [30,31], as we have observed with pharmacy staff claiming awareness of antibiotic misuse and knowledge to stop it while in reality committing inappropriate dispensing.

When compared to the results of a survey conducted in Greater Cairo to assess community pharmacists’ attitudes and practice toward antibiotic dispensing, our interview results showed good consistency in terms of dispensing without prescription (66.1% vs. 88.2%) and asking about problem history before dispensing (86.4% vs. 90.4%) [29]. Yet, there was a discrepancy in the level of knowledge regarding antibiotic appropriateness for common cold (35.5% vs. 89.6%). Likewise, a cross-sectional pilot study was conducted to assess the patterns of antibiotic dispensing in 36 community pharmacies in Greater Cairo [26]. The study reported that out of 1158 antibiotics dispensed during the whole study, only 23.3% were purchased without a prescription and 13.1% were recommended by pharmacists. However, it was reported that antibiotics were inappropriately dispensed for acute respiratory tract infections in 34.3% of pharmacists’ recommendations. These self-reported results, alongside our interview results, differed greatly from the non-prescription antibiotics dispensing rates shown in our SP encounters (98.3%). Such differences could be attributable to different study design (patient simulation visits vs. self-reported questionnaires), and perhaps location (Minia vs. Greater Cairo). However, the consistency of our self-reported interview results with these studies rules out location-related differences. Our study’s large number of randomly chosen pharmacies and SP study design with lower liability to social-desirability bias, together suggest that our SP results be more accurate in reflecting the alarming reality of antibiotic misuse in Egyptian community pharmacies. Based on these results, we believe antibiotic misuse rates in Egypt have been severely underestimated, while in fact they have reached epidemic proportions.

Reports from many countries have shown similarly high dispensing rates such as in Syria (87%), Ethiopia (93.5%), Guatemala and Mexico (80%), and India (94%) [32,33,34,35]. However, other countries have shown significantly lower dispensing rates, such as Spain (18.8%) and Zimbabwe (8.2%) [36,37]. One of the factors that could explain such variance in dispensing rates is the status of dispensing regulations and their enforcement across countries [19,38]. In an exploratory survey conducted in Ethiopia, pharmacy professionals deemed weak regulatory mechanism as one of the main reasons for non-prescription antibiotics dispensing [19]. Although most community pharmacies in Saudi Arabia are staffed by Egyptian pharmacists, a previous report showed relatively lower dispensing rates (77.3%) than observed in our SP results (98.3%) in similar SP scenarios [18]. This could be attributable to differences in pharmacy practice legislation (as in Saudi Arabia, the pharmacist is prohibited from dispensing any medication without a prescription issued by a licensed physician, excluding medications specified by the Ministry) [39,40]. In Zimbabwe (where dispensing rates were clearly low), pharmacists reported that they avoid dispensing antibiotics without prescription for fear of losing their community pharmacy license [37]. In a retrospective analysis of antibiotic sales in Chile, a significant reduction in antibiotics consumption was reported after regulatory measures taken by The Chilean Ministry of Health [41]. Therefore, an immediate step to reduce the high dispensing rates in Egypt would be to implement strict, enforceable legislation to require antibiotics to be dispensed only with an authentic prescription from a qualified physician. This step would need to go hand in hand with the development of context-specific guidelines for physicians on appropriate prescription of antibiotics for certain diagnoses, as some reports confirmed overprescribing to be a more common practice than overdispensing [42,43].

Another factor to consider in order to explain the high dispensing rates by community pharmacies is the population’s attitudes toward antibiotics and the role that pharmacy staff play in dispensing. According to our interview results, pharmacists considered patients’ perceptions about antibiotics and pharmacy staff one of the main factors contributing to antibiotic misuse and subsequent antibiotic resistance. In many reports from many countries, including developed ones, pharmacists often attribute non-prescription dispensing of antibiotics to the fear of failing to meet consumers’ demands and thus losing their satisfaction [17,44]. As populations in developing countries, like Egypt, have a lot of misconceptions about antibiotics [45,46] and lack awareness about antibiotic resistance [47,48], the task of abstaining form antibiotic dispensing by pharmacy staff becomes more difficult. Therefore, the role of national mass media campaigns is crucial in educating patients about the dangers of antibiotic overuse. In Spain, a recent report attributed the low dispensing rates to the recent awareness campaigns within the country [36]. Similarly, a significant reduction in antibiotic prescribing especially with children in France was reported after a nationwide campaign launched by the government under the title “Antibiotics are not automatic” [49]. The reduction in antibiotics consumption was associated with lower rates of resistance. In a study conducted in Finland, the nationwide reduction in using macrolide antibiotics resulted in a steady decline in the emergence of resistant strains to approximately half (16.5% to 8.6%) in the period between 1992 and 1996 [50].

The second main finding of our study is the suboptimal information-collection and counseling practice by pharmacy staff members. As evident from SP results, most staff members in the targeted pharmacies collected no information about SP symptoms (62.4%) nor did they ask for a prescription (96.6%). Our findings are similar to those reported in similar SP scenarios in the literature in different countries: Indonesia (unaware of symptoms: 68.5%, without a prescription: 91%), Albania (unaware of symptoms: 66.4%, without a prescription: 80%), Ethiopia (unaware of symptoms: 52.9%, without a prescription: 87.9%) and Colombia (unaware of symptoms: 80%, without a prescription: 80.3%) [33,51,52,53]. Such findings could be understood in the light of four main factors: community pharmacists’ work environments (lack of time and profitability) and staffing, patients’ attitude toward the importance of information collection and patient counseling services, and pharmacy practice legislations and pharmacy education. First, community pharmacists generally find the lack of time as a major barrier of proper counseling [54]. Moreover, as reported in several community pharmacists-based surveys, lack of employer reimbursement and lack of professional fees discourage community pharmacists from providing effective patient counseling services [55,56]. In Egypt, many community pharmacy employers measure their staff pharmacists’ efficiency by the volume of filled prescriptions and the number of sold medications. Therefore, pharmacy staff members’ role becomes dominated by dispensing and checking prescriptions at the expense of patient counseling. An outcome found to be a commonplace practice in many working environments [57]. One of the possible solutions for such problems is by encouraging pharmacists to allocate time for counseling as well as rewarding them for that time [58]. Furthermore, community pharmacy staffing remains problematic; several reports suggested the absence of qualified pharmacists as a main factor for high antibiotics dispensing rates and lack of proper information collection and counseling [59,60]. Once again, enforceable legal requirement of staffing pharmacies with qualified personnel, including most importantly licensed pharmacists to make dispensing decisions and provide counseling, becomes paramount, especially in a country such as Egypt.

Secondly, in many community pharmacists-based surveys, pharmacists consider patients’ attitudes to be one of the major barriers toward effective counseling [54,56,61]. Moreover, several population-based reports in the Middle East showed that large proportions of the participants think of pharmacists as mere vendors [62,63,64]. Such negative attitudes discourage pharmacists from initiating counseling services, especially in self-medication encounters, for fear of patients’ resistance and dissatisfaction [61,65]. Mass media campaigns could raise the population’s awareness of the pharmacists’ role in public health promotion and make the public more willing to seek pharmacists’ counseling services especially in self-medication encounters [66,67,68].

Thirdly, recognizing pharmacists’ clinical and patient-counseling roles in the national scope of practice legislation is of crucial importance. Although well recognized in most developed countries [69,70], the clinical and patient-counseling roles of pharmacists remain underrepresented in many developing countries’ legislation and practice guidelines [71]. This point is also related to developing proper pharmacy educational and training programs, the lack of which has been recognized as a major barrier to developing optimal pharmaceutical care services [72,73,74]. Well-trained pharmacists could be the first line of defense in combating antibiotic misuse [60], as we also observed in our SP encounters. In 2013, Booth et al., using a prospective, cross-sectional, mixed methods approach, showed that community pharmacists were able to deliver proper antibiotics treatment for UTIs in female patients, maintain antibiotics stewardship and reduce the GP workload in the UK [75]. Moreover, in a systematic review of pharmacist and consumer views on the role of the community pharmacy in public health, the authors concluded that confident, well-trained pharmacists should be able to provide better public health services for consumers [76].

Because of logistic concerns, only selected districts of Minia Governorate were included in the study sample with only urban areas studied. Unlike urban areas, the practices of pharmaceutical inspection are generally less active in rural areas, indicating larger numbers of non-pharmacists working and dispensing medications in community pharmacies. As a result, levels of inappropriate antibiotic dispensing are likely higher in rural areas. Furthermore, the SP visits were not audiotaped, rendering the collected data liable to recall bias. To overcome such concern, the data were collected instantly during the visit over the phone as the SP was directly telling all visit details to the data collector. Taking into account that the two scenarios’ actors were male, the staff members dispensing practice could be affected by gender bias. Finally, since the study took place only in one city, the findings might not necessarily generalize to all community pharmacies in the entire country, although our findings generally agree with those of studies conducted elsewhere.

## 4. Materials and Methods

### 4.1. Study Site and Population

The study took place in five districts of Minia Governorate in Upper Egypt. Those districts were: Abu Qirqas (population 63,264), Minia (population 256,732, the capital city of the governorate), Beni Mazar (population 67,699), Mallawi (population 152,198) and Samalut (population 96,029). Both patient simulation and interviews were conducted between August and October 2016. The interviews were conducted as soon as all the simulated patient visits were completed. We used interviews to compare actual practice, revealed through simulated patients, with self-reported practice. The study was approved by the local Research and Ethics Committee at the Faculty of Pharmacy, Minia University.

### 4.2. Sampling Criteria

A list of operating community pharmacies in the five districts was obtained from a multinational drug company marketing directory. A total of 415 community pharmacies were listed; 140 in Minia, 85 in Mallawi, 60 in Samalut, 46 in Abu Qirqas, and 84 in Beni Mazar. We excluded pharmacies located in rural areas of the previously mentioned districts due to logistic accessibility challenges. Participating pharmacies were randomly selected according to the districts’ population density so that a larger number of community pharmacies were selected in higher-population areas. If a pharmacy that was selected for participation could not be located due to address error, going out of business, or because the simulated patient was potentially known to the pharmacy staff, we replaced it with the nearest operational pharmacy. Simulated visits took place on different days and times to mimic real life scenarios.

### 4.3. Patient Simulation

Patient simulation is a widely implemented methodology in pharmacy practice research [77,78,79]. A simulated patient (SP) is a well-trained actor who enacts a real case scenario (seeking advice or medications) inside the pharmacy to test certain responses of the pharmacy staff [79].

### 4.4. Actors

Our SPs were two senior undergraduate pharmacy students. Each one performed a single SP scenario, consistently in all visited pharmacies. Using undergraduate students as simulated patients was employed in previous studies [18]. Student SPs extensively practiced details of the scenarios and role-played them with practicing community pharmacists who were part of the study team. All SP training sessions were audiotaped and reviewed to ensure the consistency of SP performance. Special care was taken to ensure that SPs did not use jargon during visits. To assess the feasibility of the study protocol, we conducted a pilot study of simulated scenarios in ten random community pharmacies in Minia district prior to the start of the study. Pilot data were later included in the analysis of the main study sample.

### 4.5. SP Scenario Details

Two cases of acute respiratory tract infections were implemented in the patient simulation scenario since antibiotic misuse in these conditions is very common worldwide [8,45]. The details of the two scenarios are shown in Table 6. An expert panel of two community pharmacists, three clinical pharmacists, and two pharmacy professors reviewed the scenarios. In both scenarios, the SP requested amoxicillin. Since both scenarios took place at each of the selected pharmacies, we separated the two scenarios by a one-month interval between each of them to avoid potential recognition of the SPs. Both scenarios were developed based on clinical guidelines published by American College of Physicians (ACP) and International Pharmaceutical Federation (FIP) [8,80].

### 4.6. Data Collection from SP Visits

The data collection form, used to record the details of SP visits, was developed based on previously published patient simulation studies [81,82,83,84]. The form collected information on pharmacy and pharmacy staff characteristics, information gathered by the pharmacy staff about the case, and dispensed medications. Data collection was completed during the visit, over the phone. The SP was acting as if he had his brother, for whom the antibiotic was requested, on the phone. The SP notified the staff member that the details of the visit would be received by the person on the phone, who was no more than a data collector receiving the data while the visit was ongoing. An advantage of this approach to data collection is that it minimizes recall bias relative to other methods in the literature [85,86]. The professional status of the participating pharmacy staff was confirmed by direct questions. Staff-client ratio was calculated to measure the availability of pharmacy staff to serve clients during the visit.

### 4.7. Face-to-Face Interviews

Follow-up interviews were designed and structured based on previously published studies [21,82,83]. Out of the 150 pharmacies visited by SPs, 100 pharmacies were randomly selected for follow-up interviews. During the interview, we collected data on pharmacy characteristics, pharmacy staff characteristics, counseling practices and specific questions on pharmacists’ attitudes with regard to antibiotics and their use. Pharmacists were asked to complete all sections of the interview whenever possible. Questions on counseling practices (e.g., collection of patient medical history, asking for prescription) were answered on a 5-point Likert scale (1 = Never, 2 = Rarely, 3 = Sometimes, 4 = Often, 5 = Always). The questions on antibiotics were also answered on a 5-point scale indicating levels of agreement (1 = totally disagree, 2 = disagree, 3 = not sure, 4 = agree, 5 = strongly agree). Pharmacy staff consent was obtained after explaining that all data provided would be kept confidential and de-identified, and would only be utilized for research purposes.

### 4.8. Data Analysis

Data were analyzed using R statistical software version 3.3.2 [87]. Descriptive statistics were calculated to describe community pharmacy and staff characteristics, staff responses to SP scenarios, and interview data.

## 5. Conclusions

Our study findings show that inappropriate dispensing of antibiotics in community pharmacies is highly prevalent in Egypt. There is an urgent need for multifaceted action to address this problem, including legislation on antibiotic dispensing by community pharmacies, nationwide public health awareness campaigns targeting both patients and healthcare professionals, and investment in pharmaceutical education and training.

## Figures and Tables

**Table 1:** Characteristics of pharmacy providers responding to simulated patient scenarios.

Pharmacy Characteristics	Acute Bronchitis Scenario *N* = 125	Common Cold Scenario *N* = 113
Providers		
Non-Pharmacists	55 (44%)	49 (43.4%)
Pharmacists ^1^	64 (51.2%)	62 (54.8%)
Refer to pharmacist	6 (4.8%)	2 (1.8%)
Provider’s Gender		
Male	83 (66.4%)	86 (76.1%)
Female	42 (33.6%)	27 (23.9%)
Provider’s Age (Means ± SD)	36.1 ± 10.4	38.1 ± 13.2
Time of visit		
Evening	96 (76.8%)	48 (42.5%)
Morning	9 (7.2%)	10 (8.9%)
Night	20 (16.0%)	55 (48.7%)
Day of visit		
Weekday	100 (80%)	86 (76.1%)
Weekend	25 (20%)	27 (23.9%)
Number of clients including the SP (Median, IQR)	(1, 1–2)	(2, 1–3)
Number of staff (Median, IQR)	(2, 1–3)	(1, 1–2)
Counseling duration in seconds (Median, IQR)	(65, 53–80)	(56, 38.8–80.3)
Waiting time in seconds (Median, IQR)	(5, 3–10)	(20, 6–60)
Staff/Client ratio ^2^	1.1	1.5

^1^ Pharmacist is a person who has formal pharmaceutical education (pharmacy students are also included). ^2^ Ratio = 1 means that there was approximately one staff member serving one client in each visit.

**Table 2:** Dispensing decisions and information gathered in simulated patient visits.

Item	Acute Bronchitis *n* = 125	Common Cold *n* = 113
Providers who collected relevant information before dispensing, *n* (%)	46 (36.8)	42 (37.2)
Dispensed antibiotics? *n* (%)	122 (97.6)	112 (99.1)
**Dispensed other medications? *n* (%)**		
Cough preparation and expectorant	5 (4)	0 (0.0)
Bronchodilator and mucolytic	2 (1.6)	0 (0.0)
Analgesic and antipyretic with mucolytic	1 (0.8)	0 (0.0)
Analgesic and decongestant	0 (0.0)	3 (2.7)
Analgesic & antipyretic	0 (0.0)	1 (0.9)
**Information provider collected *n* (%)**		
Doctor visit or prescription	4 (3.2)	4 (3.5)
Problem history or recurrence	1 (0.8)	0 (0.0)
Symptoms	44 (35.2)	41 (36.3)
Drug allergy	2 (1.6)	1 (0.9)
Symptoms duration	1 (0.8)	0 (0.0)

**Table 3:** Pharmacy characteristics and pharmacy staff characteristics from interview data, *N* = 83.

Community Pharmacy Characteristics	*N* (%)
**Pharmacy Type** Near to health facility Far from health facility	57 (68.7%) 26 (31.3%)
**Pharmacy ownership** Pharmacist Non-pharmacist Joint business	78 (94%) 1 (1.2%) 4 (4.8%)
**Staff responsible for patient counseling services** Pharmacist only Pharmacy assistant only Pharmacist and Pharmacy student Pharmacist and Pharmacy assistant All staff members	42 (50.6%) 2 (2.4%) 2 (2.4%) 26 (31.3%) 11 (13.2%)
Pharmacists always available in pharmacy working hours*	51 (62.2%)
Non-pharmacists always available in pharmacy working hours*	41 (50%)
**Pharmacy staff characteristics**	***N* (%)**
**Gender** Male Female	56 (67.5%) 27 (32.5%)
Age (mean ± SD)	36.7 ± 12.9
**Education** BSc in pharmaceutical sciences Non-pharmaceutical education	59 (61.1%) 24 (28.9%)
**Work Experience** Community pharmacy only Hospital pharmacy Pharmaceutical companies All	63 (75.9%) 11 (13.3%) 6 (7.2%) 3 (3.6%)
Working experience in community pharmacy in years (Median, IQR)	(10, 4–17.5)
Daily working hours inside community pharmacies in hours (Median, IQR)	(8, 6–10)

**Table 4:** Pharmacy staff self-reported information-gathering practices before antibiotic dispensing.

Item	Acute Bronchitis *n* = 125	Common Cold *n* = 113	Interviews *n* = 59
**Prescription or doctor visit**	4 (3.2%)	4 (3.5%)	20 (33.9%)
**Symptoms**	44 (35.2%)	41 (36.3%)	39 (66.1%)
**Symptoms duration**	0 (0%)	0 (0%)	30 (50.8%)
**Problem history**	1 (0.8%)	0 (0%)	51 (86.4%)

**Table 5:** Statements about antibiotics knowledge and attitude of pharmacy staff (*n* = 59).

**1. Antibiotic is essential and effective in common cold and acute bronchitis infections:**		
Agree/Strongly Agree	Not Sure	Disagree\Strongly disagree
21 (35.5%)	10 (17%)	28 (47.5%)
2. Antimicrobial resistance is a major public health threat facing the Egyptian community *		
Agree/Strongly Agree	Not Sure	Disagree\Strongly disagree
48 (96%)	1 (2%)	1 (2%)
3. Contributing factors of antibiotic misuse and antimicrobial resistance *		
I. Irrational dispensing or prescribing		32 (64%)
II. Patient culture and perceptions about antibiotics and pharmacy staff		11 (22%)
III. Self-medication		5 (10%)
IV. Improper manufacturing		1 (2%)
V. Pharmacy education		1 (2%)

* *n* = 50 as there are nine missing values.

**Table 6:** Details of simulated patient scenarios.

**Scenario details**	
The SP entered the pharmacy with a phone and approached the counter, telling the first person he saw at the pharmacy: “My elder brother is on the phone and he needs amoxicillin, please.” The pharmacy staff was informed that all information provided will be collected and recorded by the patient—the elder brother—who requested the drug on the phone. All questions asked by the pharmacy staff were answered according to the patient characteristics listed in the table. No information was provided to pharmacy staff until asked. If a question is asked outside the scenario, the answer will be “not sure.” If no questions were asked by pharmacy staff, SP was instructed to provoke the pharmacy staff by asking about: drug dosing, doses frequency, timing of doses with food, treatment duration, and drug compatibility with enalapril and hydrochlorothiazide.	
**Patient characteristics**	
**Common cold scenario (CC)**	**Acute bronchitis scenario (AB)**
The patient is the elder brother of the simulated client. He is 40 years old, male. He suffers from sneezing, rhinorrhea, sore throat, cough, low-grade fever (38 °C), headache and malaise (general discomfort) for three days. The patient also suffers from hypertension and he is on enalapril + hydrochlorothiazide. He has not tried any medications for his symptoms. He has a history of penicillin allergy. The patient has neither a previous diagnosis by a healthcare provider nor a prescription. He thinks that he has the common cold and he was told that antibiotics are effective in such conditions.	The patient is the elder brother of the simulated client. He is 40 years old, male. He suffers from productive cough, low-grade fever (38 °C), purulent green sputum, and malaise (general discomfort). The symptoms have persisted for three days. The patient also suffers from hypertension and he is on enalapril + hydrochlorothiazide. He has not tried any medications for his symptoms. He has a history of penicillin allergy. The patient has neither a previous diagnosis by a healthcare provider nor a prescription. He confirmed that he has had this problem before and the antibiotic was very effective.
